# Lactoferrin and Immunoglobulin Concentrations in Milk of Gestational Diabetic Mothers

**DOI:** 10.3390/nu13030818

**Published:** 2021-03-02

**Authors:** Jolanta Lis-Kuberka, Marta Berghausen-Mazur, Magdalena Orczyk-Pawiłowicz

**Affiliations:** 1 Department of Chemistry and Immunochemistry, Wroclaw Medical University, M. Skłodowskiej-Curie 48/50, 50-369 Wrocław, Poland; 2 Department of Pediatrics, Division of Neonatology, Wroclaw Medical University, Bartla 5, 51-618 Wroclaw, Poland; marbemaz@gmail.com

**Keywords:** breastfeeding, lactoferrin, immunoglobulins, gestational diabetes mellitus, lactation, child nutrition, human milk

## Abstract

Gestational diabetes mellitus (GDM) is associated with an increased risk of having a high-care newborn and has an impact on maternal wellbeing. This study aimed to assess the effect of GDM on the lactoferrin (LF), secretory immunoglobulin A (SIgA), immunoglobulin G (IgG), and immunoglobulin M (IgM) concentrations in early colostrum, colostrum, and transitional milk samples of hyperglycemic (*n* = 53) and normoglycemic (*n* = 49) mothers using enzyme-linked immunosorbent assay (ELISA). The concentrations of milk lactoferrin and SIgA, but not IgG and IgM, from hyperglycemic and normoglycemic mothers, showed a similar negative correlation with lactation from the first to the fifteenth day. Apart from early colostral IgG, there were no differences in concentrations of LF and immunoglobulins in milk from hyperglycemic and normoglycemic mothers. For hyperglycemia compensated by diet (GDM G1) or insulin treatment (GDM G2), slight differences were seen for LF and IgG, but not for SIgA and IgM, during an early stage of lactation only. Early colostral IgG and colostral LF of insulin-treated mothers were higher (10.01 ± 4.48 mg/L and 11.50 ± 0.58 g/L, respectively) than for diet-control diabetic mothers (7.65 ± 5.67 mg/L and 8.05 ± 1.38 g/L, respectively). GDM of mothers does not have a significant impact on immunological quality of early milk.

## 1. Introduction

Diabetes is the most common metabolic disorder complicating pregnancy, and its prevalence in Europe is estimated at ~6% with a dynamic upward trend [1]. However, the International Association of Diabetes and Pregnancy Study Groups (IADPSG) and the World Health Organization (WHO) pointed out that in certain populations gestational diabetes mellitus (GDM) might affect more than 20% of pregnancies [2,3]. The discrepancies are caused by differences in diagnostic criteria as well as screening tests used [3,4]. The lack of or insufficient glycemic control of pregnant women has an impact on higher mortality during the perinatal period [5], while appropriate meticulous antenatal care significantly improves both maternal and perinatal outcomes [6,7].

In the pathology of diabetes, an important player is inflammation [8], which may not only reduce insulin sensitivity and insulin secretion from pancreatic islets, but may also partially activate the components of innate immunity [9]. Moreover, the cytokine-induced acute phase response (also called low-grade inflammation) may interfere with the normal function of immune cells [10,11]. The rate and course of infections in mothers with gestational diabetes are higher than in healthy pregnant women, and are also reflected in concentrations of immunological parameters [12]. Pregnancy complicated by GDM is associated with an increased risk of having a high-care child, but it also has a significant impact on maternal wellbeing [6,13,14]. The short- and long-term complications for the mothers include increased incidence of maternal pre-eclampsia/eclampsia [14], hypertension [15], obstetric intervention (prolonged labor, cesarean birth, surgical complications) [16], development of type 2 diabetes (T2DM) later in life [17,18], cardiovascular disease [14], and obesity in later life [19]. On the other hand, the major morbidities and mortality amongst infants of diabetic mothers include neonatal macrosomia [20,21], congenital malformations [22,23], shoulder dystocia, higher body fat, hyperbilirubinemia, respiratory and cardiac disorders [24], hypoglycemia [25], and overweight or obesity in childhood [26,27,28,29,30].

After birth, newborns have an immature immune system [31,32,33]. Feeding maternal milk is considered as a gold standard of early postnatal nutrition and provides a valuable source of a wide range of innate and adaptive components and bioactive molecules for term and preterm newborns [34,35,36]. The major immune components delivered with maternal milk are lactoferrin (LF) and secretory immunoglobulin A (SIgA) [37,38,39,40]. Both molecules effectively support the immature newborn’s immune system and protect against colonization of gastrointestinal and respiratory tracts by pathogens, reducing the incidence of respiratory infections, sepsis, necrotizing enterocolitis, and diarrhea [41,42,43,44,45,46,47]. However, the mechanisms of biological action are different.

Lactoferrin, secreted by neutrophils and the glandular epithelial cells [48,49,50], substantially supports the innate immune system of breastfed newborns, and might protect against Gram-positive and Gram-negative bacteria, as well as both types of viruses (DNA and RNA) [51]. The mechanism of the antibacterial activity of LF relies on limiting the access of microorganisms to iron or due to its high cationic charge unfavorable modification of their cell membranes [49,50,52,53]. Moreover, due to the presence of intestinal receptors for lactoferrin [54], after endocytosis, lactoferrin can enter the nucleus and has the ability to regulate transcription of genes such as transforming growth factor β1 (TGF-β1) [55].

Of all the immunoglobulins that are present in human milk, SIgA predominates and represents over 90% of milk antibodies. Secretory IgA is mainly produced by the alveolar epithelial cells of the mammary gland [56], but a minor fraction comes, by transudation, from serum [57,58]. Milk SIgA takes part in the protection of epithelial cells of newborns and infants against invasion by pathogens, namely providing anti-pathogen effects by intracellular neutralization of viruses, inhibition of pathogen adhesion, and agglutination of viruses and bacteria [46,59,60,61,62]. Additionally, SIgA, due to the interaction with M cells, protects the intestinal surface against translocation of pathogenic bacteria [63]. Milk immunoglobulins transferred to newborns during breastfeeding participate in the shaping of the newborn’s gut microbiome. Nevertheless, the specificity of transferred immunoglobulins is limited to the range of pathogens with which the mother’s immune system has come into contact earlier [64,65]. Although the concentrations of immunoglobulin G and M (IgG and IgM) in maternal milk are significantly lower, both support the protective and modulatory actions of SIgA [40,66,67]. Taken together, the maternal milk-delivered fraction of immunoglobulins provides passive immunity to the infant [49] and is one of the crucial factors by which lactating mothers can protect breastfed newborns from a wide range of pathogens [46].

The concentrations of LF and SIgA in human milk are associated with the lactation stage [39,68,69,70,71,72]. The LF concentration in term milk was 3.16 g/L for colostrum in the postpartum period of 0–7 days, 1.73 g/L for transitional milk 8–14 days postpartum, and 0.90 g/L for mature milk >14 days postpartum [71]. Slightly higher values for term milk LF from the second and fifth week of lactation were reported by Brodhaust and coworkers [67], that is 2.61 ± 0.37 and 1.65 ± 0.18 g/L, respectively. Similar to LF, the highest concentration of term milk SIgA was reported for early colostrum from 2 to 5 days, ~2.5 g/L, then it decreased and stayed at a relatively stable level throughout transitional (8–14 days), ~1 to 0.68 g/L, to mature milk (26–35 days), ~0.7 to ~0.45 g/L [70]. The latest findings [72] confirmed that LF and SIgA concentrations were higher in early lactation and gradually decreased in later lactation. To date, the published data have shown that the levels of LF and SIgA in human milk can be associated with maternal [73] and gestational age [71], nutrient status [74,75], and parity [73]. However, it should be noted that the results of some of these studies are not consistent, mainly due to the small test group and various methods used. In contrast, the level of IgG is not affected by lactation stages in the second and fifth weeks of lactation (20 ± 2.57 and 19.1 ± 2.8 mg/L, respectively) [67,72], but early colostral IgG concentration is higher (27.85 ± 23.2 mg/L) than for late colostrum (14.89 ± 12.5 mg/L) [76]. The presence of IgM was confirmed for a small percentage of term colostrum at the level of 14 mg/L [77]. The latest findings reveal a decrease in the IgM level [72] from ~220 mg/L at days 2–5, to ~80 mg/L in the second week and ~30 mg/L in the sixth month of lactation. A similar trend but for late lactation was reported by Abuidhail and coworkers [78], namely 103 ± 31 mg/L for the first month of lactation to 48 ± 18 mg/L for the sixth month. However, Berdi and coworkers [79] reported lower levels of milk IgM (10–100 µg/L) collected from the second to the sixth day of lactation.

Collective and detailed analysis of immunoglobulins—SIgA, IgG, IgM—and lactoferrin concentrations in milk of gestational diabetic mothers is not available. A recent meta-analysis showed that available biochemical details are fragmentary and do not take into account individual immune components of maternal milk, stages of lactation, or the gestational age, and the majority are based on a small number of analyzed samples [80]. To address the gap in the profile of the main immunological proteins in milk of mothers with gestational diabetes mellitus, the aim of our study was to determine whether the levels of immunoglobulins, as well as lactoferrin, are affected by the mother’s hyperglycemia. In the current study, LF, SIgA, IgG, and IgM concentrations of milk of hyperglycemic and normoglycemic mothers, during the first 2 weeks of milk maturation, i.e., early colostrum, colostrum and transitional milk, were measured by a highly specific and sensitive method, i.e., enzyme-linked immunosorbent assay (ELISA).

## 2. Materials and Methods

### 2.1. Recruitment of Breastfeeding Mothers

For the study, lactating healthy, normoglycemic mothers (normoglycemic group) and mothers with GDM who received postpartum care after childbirth in the First Department of Gynaecology and Obstetrics located in Wroclaw Medical University (Wroclaw, Poland) were included. Lactating women were recruited for research on the basis of the consent issued by the Ethics Committee at Wrocław Medical University (No KB-882/2019) and informed written consent from all lactating mothers was received.

The mother’s age, pregnancy weight, pre-pregnancy BMI, health status, concomitant medications, parity, mode of delivery, birth weight, gestational age, infections during pregnancy, newborn’s gender and overall condition (Apgar score), and medications during lactation were recorded. For all mothers, alcohol use and cigarette smoking were exclusion criteria.

The criteria for selecting mothers with gestational diabetes mellitus were fasting blood glucose level and/or an abnormal oral glucose tolerance test (OGTT) following ingestion of 75 g of glucose. The diagnosis was based on a venous plasma fasting glucose level of more than 90 mg/dL and/or a level of glucose after ingestion of a 75-g glucose solution: ≥180 mg/dL after 60 min and/or a level of 153–199 mg/dL after 120 min [81,82,83].

The group of breastfeeding mothers with GDM was divided into two groups: Women with diet-controlled GDM (group GDM G1) and with diet- and insulin-controlled GDM (group GDM G2). The criteria for group G1 included diet and physical activity, which were sufficient to fully compensate for glucose disorders during pregnancy. However, if the postprandial value of glucose after 1 h was higher than 140 mg/dL despite the diet and physical activity, to fully correct the glucose disorders during pregnancy, it was necessary to use insulin (group GDM G2) [81,82,83].

The number of milk samples collected from healthy (normoglycemic) mothers was *n* = 49, from mothers with gestational diabetes type G1 it was *n* = 29, and from mothers with gestational diabetes type G2 it was *n* = 24.

### 2.2. Milk Collection

Milk samples were collected by 86 breastfeeding mothers at the First Department and Clinic of Gynaecology and Obstetrics, Wroclaw Medical University. Lactating mothers, after breakfast, expressed milk in a fixed period of time, between 08:00 and 12:00. Immediately after collection, the milk samples were frozen at −20 °C.

### 2.3. Sample Pre-Treatment for Analysis

Prior to assay, in order to get rid of fat and cells that interfere with the analysis, all collected milk samples were centrifuged at 3500× *g* at 4 °C for 35 min. The obtained defatted milk samples (aqueous phase) were stored at −20 °C.

### 2.4. Determination of Lactoferrin Concentration

The concentration of lactoferrin in defatted milk samples was quantified by the enzyme-linked immunosorbent assay (ELISA) in accordance with a slightly modified procedure published previously [84]. For determination, 100 μL of 25,000-, 50,000-, and 100,000-fold diluted defatted milk samples and a standard preparation of lactoferrin isolated from human milk from 0.8 to 50 ng/100 μL (Sigma Aldrich, St. Louis, MO, USA) in Tris-HCl, pH = 7.5 were transferred to the wells of a microtiter plate (Nunc International, Naperville, IL, USA) and left for 2 h at 37 °C. Rabbit anti-human lactoferrin phosphatase-labeled antibodies (Jackson ImmunoResearch Europe Ltd., Ely, UK) diluted 1:10,000 in TBS with 0.05% Tween-20 were used as a detection antibody. The reaction was developed by adding a phosphatase substrate, pNPP (4-nitrophenyl phosphate), in diethanolamine-HCl buffer (pH 9.5) (SERVA, Heidelberg, Germany) for 15 min at 37 °C. The absorbance was measured, after stopping the enzymatic reaction with 1 M NaOH, using a Synergy LX Multi-Mode Reader (BioTek Instruments, Inc., Vermont, USA). The background absorbance was measured at 405 nm with a reference filter at 630 nm, and a range of 0.035–0.039 AU when TBS was used instead of analyzed defatted milk samples or lactoferrin standard.

All defatted milk samples were assayed at three different milk sample dilutions, each in duplicate. The intra-assay and inter-assay coefficients of variation were 2.8% and 7.3%, respectively.

### 2.5. Determination of SIgA, IgG, and IgM Concentrations

The concentrations of all analyzed immunoglobulins—SIgA, IgG, and IgM—in defatted milk samples were quantified by the enzyme-linked immunosorbent assay (ELISA) previously developed with slight modification [40,76,84]. In short, for all determinations, microtiter plates (Nunc International, Naperville, IL, USA) were used. For blocking and washing steps, TBS (pH 7.5) containing 0.5% Tween-20 and TBS (pH 7.5) containing 0.05% Tween-20 were used, respectively. The antibodies used in the tests were as follows: For IgG F(ab’)2, fragments of goat anti-human IgG, for IgM rabbit anti-human IgM antibody (both from Jackson ImmunoResearch, Europe Ltd., Ely, UK), and for SIgA mouse monoclonal anti-secretory component IgA antibodies (Sigma, St. Louis, MO, USA). Standard curves were prepared using commercially available standards of human serum IgG and human IgM (both from Jackson ImmunoResearch, Europe Ltd., Ely, UK) and human colostrum IgA (Sigma, St. Louis, MO, USA), respectively. For detection, alkaline phosphatase (AP) and horseradish peroxidase (HRP)-labeled antibodies were used, for IgG rabbit anti-human IgG Fcγ fragment-specific antibodies phosphatase-labeled, for IgM goat anti-human IgM antibodies horseradish peroxidase-labeled (both from Jackson ImmunoResearch, Europe Ltd., Ely, UK), and for SIgA goat anti-mouse IgG antibodies horseradish peroxidase-labeled (Sigma, St. Louis, MO, USA). The enzymatic reactions for AP and HRP were developed with appropriate substrates and then the obtained absorbances were quantified at 405 nm (reference filter 630 nm) for AP and at 492 nm (reference filter 630 nm) for HRP, respectively, using Synergy LX Multi-Mode Reader (BioTek Instruments, Inc., Vermont, USA).

All defatted milk samples were quantified at three different sample dilutions dedicated to the individual parameter, namely 10,000-, 25,000-, and 50,000-fold diluted for SIgA, 500-, 1000-, and 1500-fold diluted for IgG and 250-, 500-, and 1000-fold diluted for IgM, each in duplicate.

The details concerning the precision of the tests performed, the coefficients of variation for intra- and inter-assay, are as follows: For SIgA-ELISA 5.0% and 7.5%, respectively, for IgG-ELISA 0.8% and 3.9%, respectively, and for IgM-ELISA 1.7% and 6.4%, respectively.

### 2.6. Statistical Analysis

The statistical analysis was done with TIBCO STATISTICA ver. 13.3 (StatSoft, Inc., Tulsa, OK, USA). The values were given as the mean ± SD (standard deviation) and additionally as the median with the twenty-fifth to seventy-fifth percentiles. The chi-square test was used to compare the study population data. For analysis, nonparametric tests were used, since large interindividual differences are common in the biochemical profile of milk. The Mann–Whitney U test was used for the calculation of statistical significance. The correlations between analyzed groups were estimated according to Spearman. A two-tailed *p*-value lower than 0.05 was regarded as significant.

## 3. Results

### 3.1. Characteristics of the Study Population

One-hundred and two milk samples were provided by breastfeeding gestational diabetic (*n* = 49) and healthy (normal glycemic) mothers (*n* = 37). The detailed characteristics of women with GDM and non-GDM are shown in Table 1. The maternal age was 33.8 ± 4.5, 33.3 ± 4.4, and 32.5 ± 4.7 years, and maternal pre-pregnancy BMI was 24.7 ± 5.3, 27.5 ± 4.9, and 22.7 ± 3.5 kg/m^2^ (*p* < 0.04), respectively, for the group of mothers with GDM G1, GDM G2, and non-GDM (non-diabetic). In the present study, more than half of mothers delivered newborns at term, at 38–41 weeks of gestation (GDM G1: 64.0%, GDM G2: 52.2%, and non-GDM: 66.7%). Moreover, none of the mothers gave birth extremely prematurely. The majority of pregnancies had been ended by elective or emergency cesarean section (GDM G1: 64.0%, GDM G2: 73.9%, and non-GDM: 78.1%) (Table 1). Primiparous mothers accounted for half of each group examined. Moreover, during pregnancy, the participants of the study suffered from hypothyroidism (28.0%, 26%.1%, and 18.9%, respectively, for GDM G1, GDM G2, and non-GDM groups) and/or genital and urinary tract infection (GDM G1: 24.0% and 16.0%, GDM G2: 13.6% and 13.6%, and non-GDM: 13.5% and 13.5%). The majority of mothers participating in the study did not report the use of medication during lactation, 72.0%, 73.9%, and 81.1%, respectively, for GDM G1, GDM G2, or non-GDM groups (Table 1).

The study found that newborns’ birth weight did not differ significantly among analyzed groups, 2883.0 ± 758.6 g, 2775.6 ± 758.1 g, and 2956.4 ± 879.5 g, respectively, and for approximately two-thirds of newborns, a good overall physical state, demonstrated by an Apgar score in the range 8–10, was defined, 66.7%, 72.7%, and 72.2%, respectively, for GDM G1, GDM G2, and non-GDM groups (Table 1).

### 3.2. Concentration of Lactoferrin

**Figure 1:**
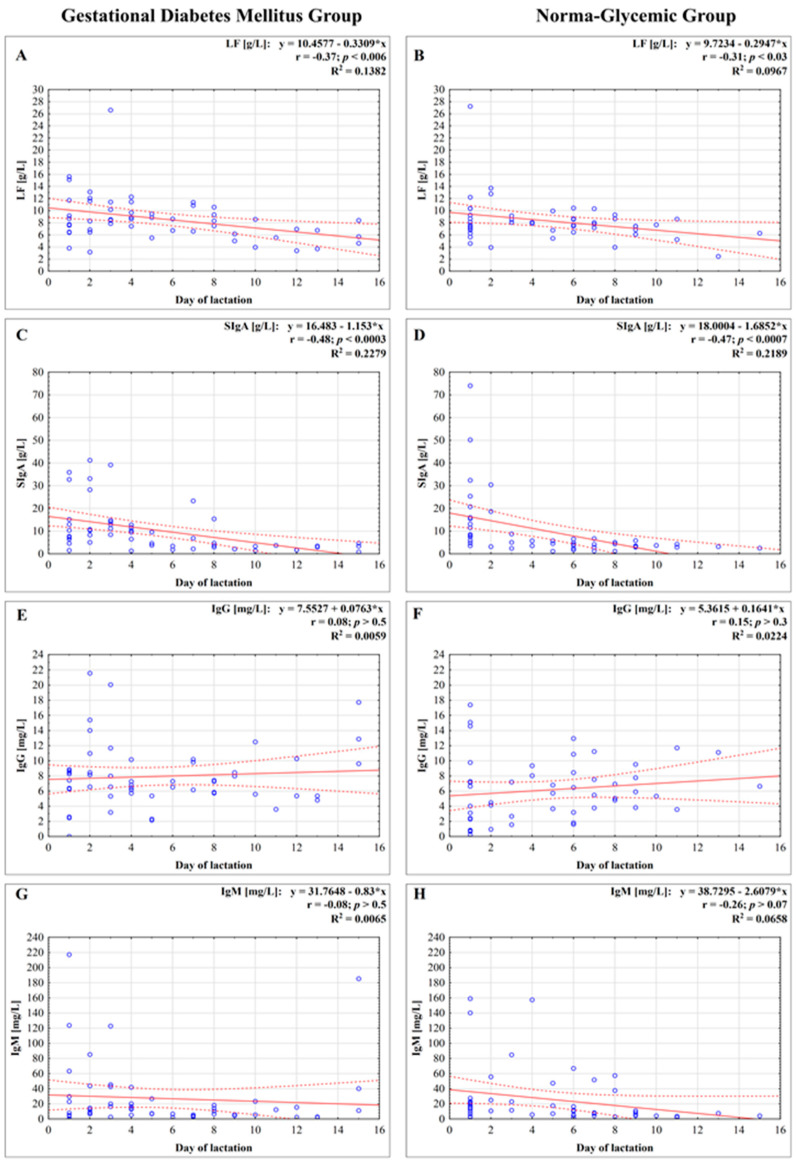
Concentration of lactoferrin (**A**,**B**), secretory immunoglobulin A (SIgA) (**C**,**D**), immunoglobulin G (IgG) (**E**,**F**), and immunoglobulin M (IgM) (**G**,**H**) in milk in the first two weeks of lactation from mothers with gestational diabetes mellitus (GDM) and normoglycemic mothers. A solid line indicates linear regression, and 95% confidence intervals are shown by dotted lines; blue circles refer to individual samples. The correlation coefficient (*r*) was calculated according to Spearman, and a *p*-value lower than 0.05 was regarded as significant. The *r*-square value is the square of the correlation coefficient. NS, not significant.

The concentration of lactoferrin in milk, for both groups, namely for mothers with GDM and non-GDM mothers, showed a weak negative correlation with the early stage of milk maturation from the first to the fifteenth day (*r* = −0.37; *p* < 0.006, and *r* = −0.31; *p* < 0.03, respectively) (Figure 1A,B).

**Figure 2:**
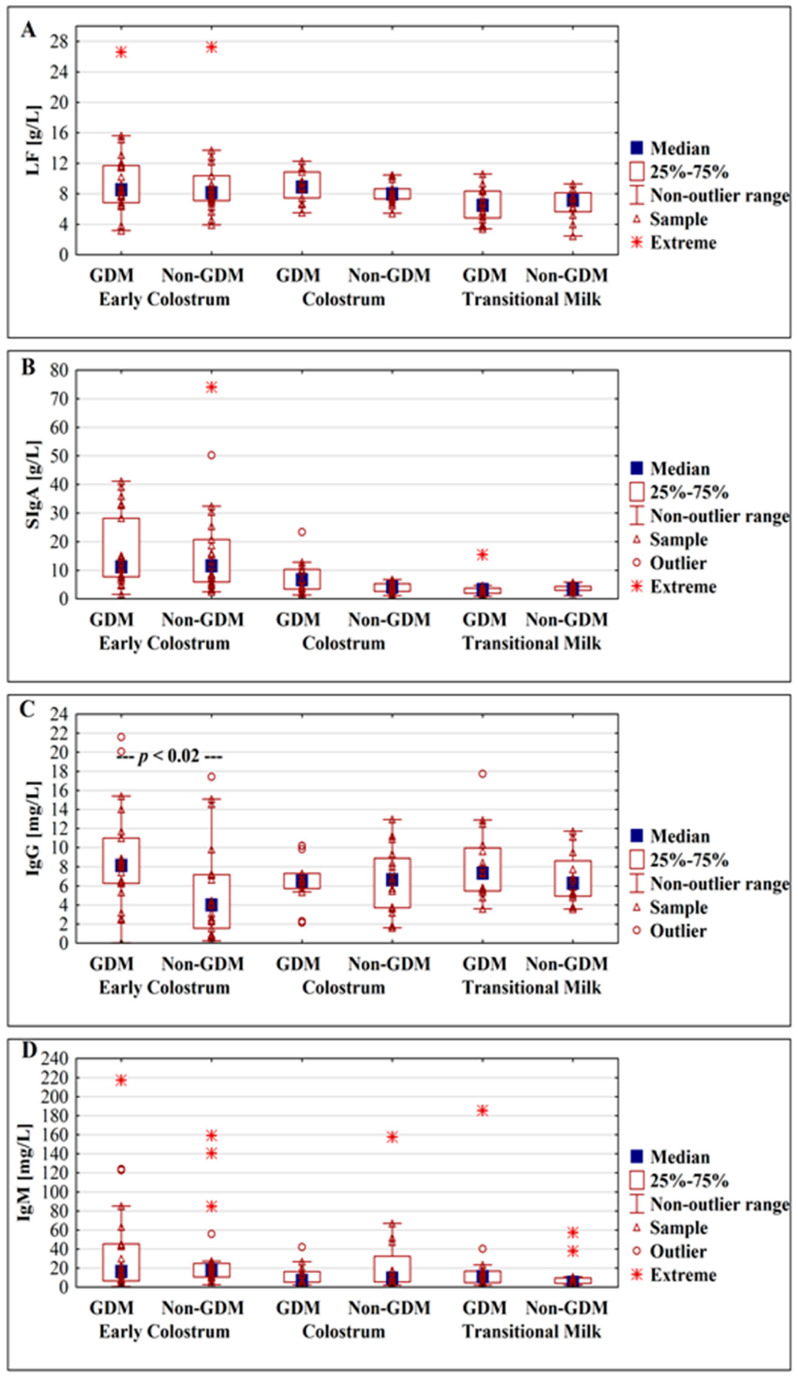
Box plot illustrating the change in concentrations of lactoferrin (**A**), SIgA (**B**), IgG (**C**), and IgM (**D**) in milk from gestational diabetic and normoglycemic (non-GDM) mothers during milk maturation from early colostrum to the transitional milk.

The concentrations of lactoferrin were analyzed at three successive lactation stages: Early colostrum, colostrum, and transitional milk. No statistically significant differences between the GDM group and non-GDM group were observed (Figure 2A). For the early colostrum from days 1–3, the mean concentration of lactoferrin was at a similar level for all analyzed groups, namely two subgroups of gestational diabetes mellitus GDM G1, GDM G2, and non-GDM, and reached values 10.02 ± 3.23, 9.67 ± 6.56, and 9.30 ± 4.81 g/L, respectively. In the next analyzed period of lactation—colostrum from days 4–7 of lactation—the concentration of lactoferrin slightly decreased, although not significantly, for GDM G1 and non-GDM groups to reach 8.05 ± 1.38 and 8.09 ± 1.37 g/L, while for GDM G2, the concentration of lactoferrin nonsignificantly increased to reach 11.50 ± 0.58 g/L. Additionally, for the colostrum group, significant differences in relation to the mother’s health condition for the GDM G2 vs. non-GDM group (11.50 ± 0.58 vs. 8.09 ± 1.37 g/L; *p* < 0.0004, respectively) and the GDM G1 vs. GDM G2 group (8.05 ± 1.38 vs. 11.50 ± 0.58 g/L; *p* < 0.001, respectively) were observed (Table 2).

After conversion of colostrum to the transitional milk, a significant decrease in the lactoferrin concentration to the value 6.45 ± 2.12 g/L (*p* < 0.001) for GDM G2 was observed, while for GDM G1 and non-GDM a nonsignificant decrease to the values of 6.71 ± 2.37 and 6.67 ± 2.01 g/L, respectively, was observed (Table 2).

### 3.3. Concentration of Immunoglobulins

#### 3.3.1. Concentration of Secretory Immunoglobulin A

The concentration of SIgA in milk showed a strong negative correlation with the early stage of milk maturation from the first to the fifteenth day, r = −0.48; *p* < 0.0003 for the GDM group as well as for the non-GDM group, r = −0.47; *p* < 0.0007, respectively (Figure 1C,D).

The concentrations of SIgA were analyzed at three successive lactation stages: Early colostrum, colostrum, and transitional milk. No statistically significant differences between GDM and non-GDM groups were observed (Figure 2B).

The mean concentration of SIgA was the highest (17.45 ± 10.86, 14.59 ± 14.06, and 17.44 ± 17.63 g/L) for all the analyzed groups, GDM G1, GDM G2, and non-GDM, in early colostrum from the first to the third day. For GDM G1 and non-GDM groups, in comparison to early colostrum, a significant decrease in SIgA concentration in the group of colostrum obtained from the fourth to seventh days of lactation was observed, and the value reached 6.24 ± 3.98 (*p* < 0.001) and 3.98 ± 1.75 g/L (*p* < 0.00006), respectively (Table 2).

In the subsequent period of lactation in milk from the eighth to fifteenth days for all analyzed groups, the mean value of SIgA concentration was insignificantly lower, 3.23 ± 1.30 for GDM G1, 3.92 ± 4.19 for GDM G2, and 3.56 ± 1.24 g/L for non-GDM, and no significant differences were observed in relation to the mother’s health condition (Table 2).

#### 3.3.2. Concentration of IgG

The concentration of IgG, for both groups, in milk of mothers with GDM and non-GDM, showed no correlation with the early stage of milk maturation from the first to the fifteenth day (*r* = 0.08; *p* > 0.5, and *r* = 0.15; *p* > 0.3, respectively) (Figure 1E,F).

A significant difference between GDM and non-GDM was observed in analyzed early colostrum groups from days 1–3 of lactation (*p* < 0.02), but not for groups of colostrum from days 4–7 and transitional milk from days 8–15 of lactation (Figure 2C).

The mean concentrations of IgG in early colostrum from mothers with GDM G1, GDM G2, and non-GDM were 7.65 ± 5.67, 10.01 ± 4.48, and 5.40 ± 5.07 mg/L, respectively, and a significant difference was observed in relation to GDM G2 vs. non-GDM (*p* < 0.008) (Table 2). The mean concentrations of IgG in milk remained at an almost unchanged level in the next analyzed period of lactation: For colostrum from days 4–7 (6.31 ± 2.66, 7.33 ± 1.94, and 6.69 ± 3.38 mg/L) and transitional milk from days 8–15 of lactation (7.96 ± 5.18, 8.27 ± 2.83, and 6.85 ± 2.70 mg/L) for GDM G1, GDM G2, and non-GDM groups, respectively (Table 2).

#### 3.3.3. Concentration of IgM

The concentration of IgM in milk from GDM and non-GDM mothers showed no correlation with the early stage of milk maturation from the first to the fifteenth day (*r* = 0.08, *p* > 0.5 and *r* = −0.26, *p* > 0.07, respectively) (Figure 1G,H). In analyzed periods of lactation for GDM vs. non-GDM groups no significant differences were observed (Figure 2D).

In the first three days of lactation, the concentration of IgM was the highest and reached 49.75 ± 67.31, 26.26 ± 21.19, and 32.86 ± 43.21 mg/L, and in the subsequent stage, i.e., colostrum from days 4–7 of lactation, it decreased to the values 13.44 ± 12.77, 9.71 ± 5.96, and 26.54 ± 40.04 mg/L for groups GDM G1, GDM G2, and non-GDM, respectively. In the group of milk from the eighth to fifteenth day of lactation a decrease in IgM concentration to the values 13.08 ± 13.75 and 12.44 ± 17.08 mg/L for GDM G1 and non-GDM groups, respectively, was observed, while for the GDM G2 group an increase was recorded (28.19 ± 55.64 mg/L) (Table 2).

## 4. Discussion

According to the scientific databases, namely the National Center for Biotechnology Information and Web of Science, our findings are the first which in detail characterize the concentration of lactoferrin and immunoglobulins of gestational diabetic mothers’ milk during early lactation, with additional emphasis on the severity of hyperglycemia.

Diabetes is a complex metabolic disease, which affects the glucose status of the human body. The high blood glucose level coupled with activation of adipocytes and macrophages in fat as well as inflammatory cytokine production generates a low-grade chronic inflammatory response, which disrupts the cellular function of blood B cells and enhances their apoptosis [8,9,85,86] resulting in higher IgA and lower IgG and IgM levels in serum of type 2 diabetic patients [12].

Breastfeeding is known to play an essential role in nutrition programming in the early stages of growth and have an impact on lowering the risk of developing diabetes and obesity in later life [87]. Maturation of maternal milk is related to dynamic changes in both nutritional and bioactive components, including immunologically important molecules such as innate and adaptive factors [38,40,59,76]. From the first to fifteenth day of lactation, the concentrations of LF and SIgA were negatively correlated with milk maturation of hyperglycemic (*r* = −0.37 and *r* = −0.48, respectively) and normoglycemic mothers (*r* = −0.31 and *r* = −0.48, respectively), and the values of coefficients were at almost the same level. In contrast, the concentrations of IgG and IgM in milk of hyperglycemic as well as normoglycemic mothers showed no relation with the early stage of milk maturation (IgG: *r* = 0.08 and *r* = 0.15, IgM: *r* = −0.08 and *r* = −0.26, respectively). The lack of significant differences in concentrations of LF and immunoglobulins is probably the result of special care and regular glycemic monitoring and treatment of pregnant women, which substantially impacts dysfunction of the immune system caused by gestational diabetes [88,89,90]. Moreover, apart from filtration from serum, the main synthesis of milk immunoglobulins by B cells takes place locally within the mammary gland [58] and seems not to be substantially affected.

In our cohort, among analyzed immunological components, only early colostral IgG of mothers with gestational diabetes was significantly higher (8.83 ± 5.08 mg/L) than in milk from normoglycemic mothers (5.40 ± 5.07 mg/L). At this point, it is worth emphasizing that outliers and the range of IgG concentration in early colostrum, the most variable stage of lactation among lactating mothers [59], are higher in comparison to the other analyzed periods and can substantially impact the results. Moreover, the mammary gland responds to the hormonal changes associated with delivery, namely placental oxidative stress and the inflammatory response, among others [91], and the stimulus of the newborn sucking to production and release of milk by the alveoli [92]. All these factors can finally translate to the early colostral level of IgG.

On the other hand, mothers’ hyperglycemia during pregnancy did not impact milk LF, SIgA, and IgM concentrations (Figure 2). Previously, Kaushik and coworkers [93] reported no differences in early colostral (first day) IgA concentration between mothers with glucose intolerance (23 ± 8 g/L) and with normal glucose level (25 ± 8 g/L). Another analysis showed that the immunoglobulin profile of early colostrum of diabetic mothers is characterized by lower levels of IgA and IgG [94,95]. However, the sensitivity and accuracy of the tests used [95], namely radial immunodiffusion, are significantly lower and currently no longer used for determination of immunoglobulin concentrations. In turn, estimation of lactoferrin and SIgA in 2-week-old milk showed that SIgA in milk of mothers with GDM was 63.6% lower than in milk of normoglycemic mothers. However, determinations were based on a semi-quantitative method [96] and need confirmation with appropriate, dedicated immunochemical methods.

An essential component of management of gestational diabetes mellitus is lifestyle change, which in the case of moderate glycemia is usually sufficient for the treatment of pregnant women [81,82,83,97]. However, when glycemic targets cannot be achieved by diet and physical activity, medications should be administered. Taking into account the severity of gestational diabetes, namely the possibility of effective glycemic control by diet (GDM G1) or insulin treatment (GDM G2), we found that LF and IgG, but not SIgA and IgM, concentrations differed between analyzed groups, but during an early stage of lactation only. The concentration of early colostral IgG of insulin-treated mothers was higher (10.01 ± 4.48 mg/L) than for diet-control diabetic mothers (7.65 ± 5.67 mg/L) and normoglycemic mothers (5.40 ± 5.07 mg/L), although not significantly. Colostral LF concentration of insulin-treated mothers was significantly higher (11.50 ± 0.58 g/L) than for diet-control diabetic mothers (8.05 ± 1.38 g/L), as well as for normoglycemic mothers (8.09 ± 1.37 g/L). In fact, the concentration of LF in milk of mothers with gestational diabetes determined in our study cannot be compared with other findings. However, the sole study, by Smilnowitz and coworkers [96], showed that in the transitional milk, as a consequence of maternal glucose dysregulation, the level of lactoferrin increased; however, data concerning the real concentration were not presented.

Providing an appropriate set of immunoglobulins along with the mother’s milk is crucial for newborns of diabetic mothers who require increased care, as well as for healthy newborns. Their action on the mucosal surface of the gastrointestinal tract is supported by LF, which acts as an immunomodulator responsible for up- and downregulation of both innate and adaptive immune cells, and participates in maintaining homeostasis [50]. Although milk immunoglobulins are exposed to a varying degree to be digested by the newborn, all milk isotypes that pass through the intestines are found in the feces of infants [98], with the most abundant being SIgA due to the secretory component crucial for higher resistance to proteolytic cleavage [47,98,99]. However, it should be emphasized that not only IgA activity is important. Milk IgG is essential for ensuring homeostasis in gut-associated lymphoid tissue due to the prevention of the activation of the immune system and shapes development of innate lymphoid cells present in the newborn’s gastrointestinal tract [100,101] might activate phagocytes, and calm the increased reaction to allergens [59]. In turn, milk IgM participates in opsonization of Gram-negative bacteria [102] and in addition to milk SIgA, in removing antigens or pathogens by their agglutination [103].

The pathogen-specific IgG in milk, induced during maternal infection or immunization, might mediate the elimination of enteric bacteria and viruses, and thus protects neonates against infection [104,105,106]. Additionally, the glycans attached to milk immunoglobulins, as a link between the innate and adaptive immune systems, might be an “additional binding site” for bacterial lectin receptors, and thus decrease pathogen interaction with the newborn’s cells [76,107,108]. Moreover, milk immunoglobulin composition might be shaped by the mother’s allergic status, previous infection, and vaccination during pregnancy [47]. The latest studies reported that breast milk of mothers diagnosed with COVID-19 contains SARS-CoV-2-specific IgA and IgG, and therefore breastfed newborns and infants, by passive immunity transferred from mothers, might be protected against COVID-19 [47,109].

However, like most studies, ours also had weaknesses. In the presented results, the phenomenon of inter-individual differences in the group of mothers with diabetes as well as in the group of healthy mothers was at least partially responsible for the large differences in concentrations between individual mothers. Additionally, future studies are needed to evaluate the potential impact of socioeconomic status and even perinatal stress on immunoglobulin profile of milk expressed by diabetic mothers, since both are well-defined factors that impact the immunological quality of maternal milk [110,111]. On the other hand, the indisputable strength of the presented data is the very valuable, difficult to acquire pool of early colostrum and colostrum samples of hyperglycemic mothers with different severity levels classified as GDM G1 and GDM G2 that were collected. Moreover, a very homogeneous group of lactating mothers in terms of place of residence allowed us to avoid previously reported discrepancies caused by geographic location [71,110]. It should also be emphasized that the use of a solid phase sensitive method with highly specific antibodies guarantees high precision and reproducibility for all determinations of immunological components.

Overall, our results corroborate previous reports on the effects of diabetes on the immunoglobulin profile, but further detailed studies are required. However, one should be very careful when drawing conclusions, taking into account the very large individual differences between mothers in terms of profiles of immunologically important colostral components in our cohort of lactating mothers.

GDM is a very complex disorder with no common pathogenesis, typically involving the development of insulin resistance associated with compensatory hyperinsulinemia. Therefore, it would be beneficial to extend the future studies and look in parallel to the levels of hormonal impairment of hyperglycemic mothers. In addition, it would be of interest to have the time points for collection of transitional milk all the way to the mature milk beyond the two-week period. Moreover, the integration of the severity of hyperglycemia and duration of insulin treatment for the future study should be taken into account. However, the question of whether there are any other immunological parameters in mother milk that may show GDM-induced variability still remains unanswered. GDM is an inflammatory disorder; thus, looking into inflammatory mediators in both serum and milk would improve the value of future studies.

## 5. Conclusions

The presented results clearly show that the immunological quality of the milk of gestational diabetic mothers, expressed by the concentration of lactoferrin and immunoglobulins, is not strongly affected by gestational diabetes of mothers. However, the severity of hyperglycemia, defined by classification as GDM G1 and GDM G2, has a moderate impact only on some immunological components at an early stage of lactation. During the postnatal period, the contribution of maternal milk to the newborn and infant’s feeding is undeniable, from an immunological standpoint. The high immunological quality of milk, despite the small volume at the beginning of lactation, is extremely important, especially for newborns of mothers with diabetes. Infants of diabetic mothers are exposed to an increased risk of complications compared to infants of healthy mothers. In this context, the feeding of mother’s milk, whose immunological and immune-protective properties are similar in the initial stage of lactation, should be a priority for diabetic mothers. It is well known how high the health costs of not breastfeeding are [112], and therefore newborns of mothers with gestational diabetes should not be at increased risk. Thus, supporting breastfeeding should be one of the overarching goals in the prevention of development of type 2 diabetes among children and mothers with recent gestational diabetes mellitus, and thereby one of the pillars of public health nutrition programs around the world.

## Figures and Tables

**Table 1:** Characteristics of the study population.

	Gestational Diabetes Mellitus G1 *N* = 26 (% (*n/N*))	Gestational Diabetes Mellitus G2 *N* = 23 (% (*n/N*))	Normoglycemic Mothers *N* = 37 (% (*n/N*))	Chi-Square Test χ^2^	*p*-Value
Race/ethnicity white Europeans	100% (26/26)	100% (23/23)	100% (37/37)	-	-
Maternal age (mean ± SD) 20–29 30–34 35–40 40+	33.8 ± 4.5 20.0% (5/25) 32.0% (8/25) 40.0% (10/25) 8.0% (2/25)	33.3 ± 4.4 9.1% (2/22) 59.1% (13/22) 27.3% (6/22) 4.5% (1/22)	32.5 ± 4.7 24.3% (9/37) 35.1% (13/37) 37.8% (14/37) 2.7% (1/37)	5.78	0.45
Maternal pre‑pregnancy BMI, kg/m^2^ (mean ± SD) underweight (<18.5) normal weight (18.5–24.9) overweight (25–29.9) obesity class 1 (30–34.9) obesity class 2 (35–39.9)	24.7 ± 5.3 15.5% (3/19) 36.8% (7/19) 31.6% (6/19) 15.5% (3/19) non	27.5 ± 4.9 non 30.8% (4/13) 46.2% (6/13) 15.4% (2/13) 7.7% (1/13)	22.7 ± 3.5 3.8% (1/26) 76.9% (20/26) 15.4% (4/26) 3.8% (1/26) non	16.86	0.04
Parity 1 2 3 4	50.0% (12/24) 37.5% (9/24) 8.3% (2/24) 4.2% (1/24)	50.0% (11/22) 36.4% (8/22) 9.1% (2/22) 4.5% (1/22)	62.9% (22/35) 28.6% (10/35) 5.7% (2/35) 2.9% (1/35)	1.39	0.97
Gestational age (mean ± SD) extremely preterm: 24–27 weeks preterm: 28–35 weeks near term: 36–37 weeks term: 38–41 weeks	37.4 ± 3.1 non 23.1% (6/26) 15.4% (4/26) 64.0% (16/26)	36.8 ± 2.7 non 26.1% (6/23) 21.7% (5/23) 52.2% (12/23)	37.2 ± 3.3 non 21.2% (7/33) 12.1% (4/33) 66.7% (22/33)	1.41	0.85
Delivery mode vaginal birth cesarean section elective c-section emergency c-section	36% (9/25) 64% (16/25) 37.5% (6/16) 62.5% (10/16)	26.1% (6/23) 73.9% (17/23) 58.8% (10/17) 41.2% (7/17)	21.9% (7/32) 78.1% (25/32) 44% (11/25) 56% (14/25)	1.44	0.49
Birth weight (g) (mean ± SD) appropriate for gestational age (AGA) small for gestational age (SGA)	2883.0 ± 758.6 88.9% (24/27) 11.1% (3/27)	2775.6 ± 758.1 95.7% (22/23) 4.3% (1/23)	2956.4 ± 879.5 91.9% (34/37) 8.1% (3/37)	0.77	0.69
Newborn’s gender male female	50.0% (13/26) 50.0% (13/26)	41.7% (10/24) 58.3% (14/24)	48.6% (17/35) 51.4% (18/35)	3.59	0.17
Newborn’s overall condition (Apgar score) 8–10 4–7 <4	66.7% (18/27) 33.3% (9/27) non	72.7% (16/22) 27.3% (6/22) non	72.2% (26/36) 27.8% (10/36) non	0.29	0.87
Infections during pregnancy GBS (+) genital tract infection urinary infection other	4.0% (1/25) 24.0% (6/25) 16.0% (4/25) 28.0% (7/25)	4.5% (1/22) 13.6% (3/22) 13.6% (3/22) 4.5% (1/22)	18.9% (7/37) 13.5% (5/37) 13.5% (5/37) 13.5% (5/37)	6.76	0.35
Mother’s diseases hypertension hypothyroidism gestational cholestasis other	12.0% (3/25) 28.0% (7/25) non 4.0% (1/25)	17.4% (4/23) 26.1% (6/23) 8.7% (2/23) 4.3% (1/23)	10.8% (4/37) 18.9% (7/37) 2.7% (1/37) 8.1% (3/37)	3.36	0.77
Medicines during lactation (other than insulin) no medications thyroxine	72.0% (18/25) 28% (7/25)	73.9% (17/23) 26.1% (6/23)	81.1% (30/37) 18.9% (7/37)	0.80	0.68

The table shows values, which are given as means ± SDs (ranges) and the percentage value representing the number of milk donors in the given subgroup (*n*) in relation to all milk donors, for whom the specific information was available. “-” not analyzed.

**Table 2:** Concentrations of lactoferrin and immunoglobulins in milk from diabetic mothers during milk transformation from early colostrum to the transitional milk.

LF and Immunoglobulins	Stage of Lactation	Group			*p*-Value GDM G1 vs. Normoglycemic	*p*-Value GDM G2 vs. Normoglycemic	*p*-Value GDM G1 vs. GDM G2
		GDM G1 *n* = 29	GDM G2 *n* = 24	Normoglycemic *n* = 49			
Lactoferrin [g/L]	Early colostrum (Days 1–3) (*n* = 13/10/21)	10.02 ± 3.23 8.68 (7.59–12.07)	9.67 ± 6.56 8.14 (6.84–11.44)	9.30 ± 4.81 8.12 (7.09–10.36)	*p* > 0.38 (NS)	*p* > 0.91 (NS)	*p* > 0.44 (NS)
	Colostrum (Days 4–7) (*n* = 10/4/16)	8.05 ± 1.38 8.64 (6.76–8.89)	11.50 ± 0.58 11.42 (11.13–11.87)	8.09 ± 1.37 7.97 (7.33–8.64)	*p* > 0.81 (NS)	*p* < 0.0004	*p* < 0.001
	Transitional milk (Days 8–15) (*n* = 6/10/12)	6.71 ± 2.37 6.99 (4.63–8.58)	6.45 ± 2. 12 6.47 (5.04–7.51) *p* ^2^ < 0.001	6.67 ± 2.01 7.13 (5.65–8.15)	*p* = 1 (NS)	*p* > 0.58 (NS)	*p* > 0.79 (NS)
SIgA [g/L]	Early colostrum (Days 1–3) (*n* = 13/10/21)	17.45 ± 10.86 12.97 (10.36–28.22)	14.59 ± 14.06 9.39 (5.05–14.79)	17.44 ± 17.63 11.52 (5.93–20.78)	*p* > 0.44 (NS)	*p* > 0.63 (NS)	*p* > 0.31 (NS)
	Colostrum (Days 4–7) (*n* = 10/4/16)	6.24 ± 3.98 5.58 (3.43–9.57) *p* ^1^ < 0.001	11.37 ± 8.79 9.92 (5.90–16.84)	3.98 ± 1.75 4.05 (2.66–5.33) *p* ^1^ < 0.00006	*p* > 0.20 (NS)	*p* > 0.08 (NS)	*p* > 0.37 (NS)
	Transitional milk (Days 8–15) (*n* = 6/10/12)	3.23 ± 1.30 3.56 (2.93–3.76)	3.92 ± 4.19 2.57 (1.75–3.47)	3.56 ± 1.24 3.34 (2.94–4.33)	*p* > 0.89 (NS)	*p* > 0.28 (NS)	*p* > 0.63 (NS)
IgG [mg/L]	Early colostrum (Days 1–3) (*n* = 13/10/21)	7.65 ± 5.67 7.44 (3.20–8.85)	10.01 ± 4.48 8.36 (6.58–11.70)	5.40 ± 5.07 4.00 (1.56–7.20)	*p* > 0.15 (NS)	*p* < 0.008	*p* > 0.23 (NS)
	Colostrum (Days 4–7) (*n* = 10/4/16)	6.31 ± 2.66 6.50 (5.36–7.30)	7.33 ± 1.94 6.48 (6.15–8.50)	6.69 ± 3.38 6.65 (3.72–8.89)	*p* > 0.77 (NS)	*p* > 0.75 (NS)	*p* > 0.63 (NS)
	Transitional milk (Days 8–15) (*n* = 6/10/12)	7.96 ± 5.18 5.71 (5.37–9.62)	8.27 ± 2.83 7.71 (5.74–10.28)	6.85 ± 2.70 6.28 (4.93–8.65)	*p* > 0.89 (NS)	*p* > 0.20 (NS)	*p* > 0.56 (NS)
IgM [mg/L]	Early colostrum (Days 1–3) (*n* = 13/10/21)	49.75 ± 67.31 14.65 (6.61–85.22)	26.26 ± 21.19 21.45 (8.37–43.81)	32.86 ± 43.21 18.09 (10.67–24.92)	*p* > 0.91 (NS)	*p* > 0.98 (NS)	*p* > 0.97 (NS)
	Colostrum (Days 4–7) (*n* = 10/4/16)	13.44 ± 12.77 7.04 (5.21–20.25)	9.71 ± 5.96 9.27 (4.71–14.71)	26.54 ± 40.04 9.34 (5.52–32.54)	*p* > 0.51 (NS)	*p* > 0.61 (NS)	*p* > 0.83 (NS)
	Transitional milk (Days 8–15) (*n* = 6/10/12)	13.08 ± 13.75 8.91 (5.40–12.29)	28.19 ± 55.64 12.35 (4.25–18.22)	12.44 ± 17.08 4.99 (3.91–9.66)	*p* > 0.43 (NS)	*p* > 0.38 (NS)	*p* > 0.87 (NS)

Values are given as mean ± SD, median, and twenty-fifth–seventy-fifth percentiles in parentheses. The Mann–Whitney U test was used for statistical calculations, and a *p*-value lower than 0.05 was regarded as significant. ^1^ Significantly different from early colostrum (days 1–3). ^2^ Significantly different from colostrum (days 4–7).

## Data Availability

Due to the sensitivity of the data and the lack of consent for online posting, individual data cannot be made accessible.

## Abbreviations

GDM	gestational diabetes mellitus
GDM G1	diet-controlled GDM
GDM G2	diet- and insulin-controlled GDM
IgG	immunoglobulin G
IgM	immunoglobulin M
LF	lactoferrin
SIgA	secretory immunoglobulin A

## References

[1] Eades C.E., Cameron D.M., Evans J.M.M. (2017). Prevalence of gestational diabetes mellitus in Europe: A meta-analysis. Diabetes Res. Clin. Pract..

[2] Buckley B.S., Harreiter J., Damm P., Corcoy R., Chico A., Simmons D., Vellinga A., Dunne F., DALI Core Investigator Group (2012). Gestational diabetes mellitus in Europe: Prevalence, current screening practice and barriers to screening. A review. Diabet. Med..

[3] Egan A.M., Vellinga A., Harreiter J., Simmons D., Desoye G., Corcoy R., Adelantado J.M., Devlieger R., Van Assche A., Galjaard S. (2017). Epidemiology of gestational diabetes mellitus according to IADPSG/WHO criteria among obese pregnant women in Europe. Diabetologia.

[4] Benhalima K., Van Crombrugge P., Hanssens M., Devlieger R., Verhaeghe J., Mathieu C. (2012). Gestational diabetes: Overview of the new consensus screening strategy and diagnostic criteria. Acta Clin. Belg..

[5] Buhary B.M., Almohareb O., Aljohani N., Alzahrani S.H., Elkaissi S., Sherbeeni S., Almaghamsi A., Almalki M. (2016). Glycemic control and pregnancy outcomes in patients with diabetes in pregnancy: A retrospective study. Indian J. Endocrinol. Metab..

[6] Nilofer A.R., Raju V.S., Dakshayini B.R., Zaki S.A. (2012). Screening in high-risk group of gestational diabetes mellitus with its maternal and fetal outcomes. Indian J. Endocrinol. Metab..

[7] Kumari R., Dalal V., Kachhawa G., Sahoo I., Khadgawat R., Mahey R., Kulshrestha V., Vanamail P., Sharma J.B., Bhatla N. (2018). Maternal and perinatal outcome in gestational diabetes mellitus in a tertiary care hospital in Delhi. Indian J. Endocrinol. Metab..

[8] Tsalamandris S., Antonopoulos A.S., Oikonomou E., Papamikroulis G.A., Vogiatzi G., Papaioannou S., Deftereos S., Tousoulis D. (2019). The role of inflammation in diabetes: Current concepts and future perspectives. Eur. Cardiol..

[9] Berbudi A., Rahmadika N., Tjahjadi A.I., Ruslami R. (2020). Type diabetes and its impact on the immune system. Curr. Diabetes Rev..

[10] Donath M.Y., Shoelson S.E. (2011). Type diabetes as an inflammatory disease. Nat. Rev. Immunol..

[11] Zhong J., Gong Q., Mima A. (2017). Inflammatory regulation in diabetes and metabolic dysfunction. J. Diabetes Res..

[12] Guo X., Meng G., Liu F., Zhang Q., Liu L., Wu H., Du H., Shi H., Xia Y., Liu X. (2016). Serum levels of immunoglobulins in an adult population and their relationship with type diabetes. Diabetes Res. Clin. Pract..

[13] Buchanan T.A., Xiang A.H., Page K.A. (2012). Gestational diabetes mellitus: Risks and management during and after pregnancy. Nat. Rev. Endocrinol..

[14] Sandsæter H.L., Horn J., Rich-Edwards J.W., Haugdahl H.S. (2019). Preeclampsia, gestational diabetes and later risk of cardiovascular disease: Women’s experiences and motivation for lifestyle changes explored in focus group interviews. BMC Pregnancy Childbirth.

[15] Bryson C.L., Ioannou G.N., Rulyak S.J., Critchlow C. (2003). Association between gestational diabetes and pregnancy-induced hypertension. Am. J. Epidemiol..

[16] Metcalfe A., Sabr Y., Hutcheon J.A., Donovan L., Lyons J., Burrows J., Joseph K.S. (2017). Trends in obstetric intervention and pregnancy outcomes of canadian women with diabetes in pregnancy from to 2015. J. Endocr. Soc..

[17] Bellamy L., Casas J.P., Hingorani A.D., Williams D. (2009). Type diabetes mellitus after gestational diabetes: A systematic review and meta-analysis. Lancet.

[18] Herath H., Herath R., Wickremasinghe R. (2017). Gestational diabetes mellitus and risk of type diabetes years after the index pregnancy in Sri Lankan women-A community based retrospective cohort study. PLoS ONE.

[19] Hart C.L., Hole D.J., Lawlor D.A., Davey Smith G. (2007). How many cases of Type diabetes mellitus are due to being overweight in middle age? Evidence from the Midspan prospective cohort studies using mention of diabetes mellitus on hospital discharge or death records. Diabet. Med..

[20] Langer O., Mazze R. (1988). The relationship between large-for-gestational-age infants and glycemic control in women with gestational diabetes. Am. J. Obstet. Gynecol..

[21] Gandhi P., Bustani R., Madhuvrata P., Farrell T. (2012). Introduction of metformin for gestational diabetes mellitus in clinical practice: Has it had an impact?. Eur. J. Obstet. Gynecol. Reprod. Biol..

[22] Suhonen L., Hiilesmaa V., Teramo K. (2000). Glycaemic control during early pregnancy and fetal malformations in women with type I diabetes mellitus. Diabetologia.

[23] Guerin A., Nisenbaum R., Ray J.G. (2007). Use of maternal GHb concentration to estimate the risk of congenital anomalies in the offspring of women with prepregnancy diabetes. Diabetes Care.

[24] Mitanchez D., Yzydorczyk C., Simeoni U. (2015). What neonatal complications should the pediatrician be aware of in case of maternal gestational diabetes?. World J. Diabetes.

[25] Opara P.I., Jaja T., Onubogu U.C. (2010). Morbidity and mortality amongst infants of diabetic mothers admitted into a special care baby unit in Port Harcourt, Nigeria. Ital. J. Pediatr..

[26] Gillman M.W., Rifas-Shiman S., Berkey C.S., Field A.E., Colditz G.A. (2003). Maternal gestational diabetes, birth weight, and adolescent obesity. Pediatrics.

[27] Boney C.M., Verma A., Tucker R., Vohr B.R. (2005). Metabolic syndrome in childhood: Association with birth weight, maternal obesity, and gestational diabetes mellitus. Pediatrics.

[28] Kim S.Y., Sharma A.J., Callaghan W.M. (2012). Gestational diabetes and childhood obesity: What is the link?. Curr. Opin. Obstet. Gynecol..

[29] Bider-Canfield Z., Martinez M.P., Wang X., Yu W., Bautista M.P., Brookey J., Page K.A., Buchanan T.A., Xiang A.H. (2017). Maternal obesity, gestational diabetes, breastfeeding and childhood overweight at age years. Pediatr. Obes..

[30] Kaul P., Bowker S.L., Savu A., Yeung R.O., Donovan L.E., Ryan E.A. (2019). Association between maternal diabetes, being large for gestational age and breast-feeding on being overweight or obese in childhood. Diabetologia.

[31] Simon A.K., Hollander G.A., McMichael A. (2015). Evolution of the immune system in humans from infancy to old age. Proc. Biol. Sci..

[32] Kollmann T.R., Kampmann B., Mazmanian S.K., Marchant A., Levy O. (2017). Protecting the newborn and young infant from infectious diseases: Lessons from immune ontogeny. Immunity.

[33] Yu J.C., Khodadadi H., Malik A., Davidson B., Salles É.D.S.L., Bhatia J., Hale V.L., Baban B. (2018). Innate immunity of neonates and infants. Front. Immunol..

[34] Rubarth L.B. (2013). Infants of diabetic mothers. Neonatal Netw..

[35] Lyons K.E., Ryan C.A., Dempsey E.M., Ross R.P., Stanton C. (2020). Breast milk, a source of beneficial microbes and associated benefits for infant health. Nutrients.

[36] Martín-Álvarez E., Diaz-Castro J., Peña-Caballero M., Serrano-López L., Moreno-Fernández J., Sánchez-Martínez B., Martín-Peregrina F., Alonso-Moya M., Maldonado-Lozano J., Hurtado-Suazo J.A. (2020). Oropharyngeal colostrum positively modulates the inflammatory response in preterm neonates. Nutrients.

[37] Liao Y., Alvarado R., Phinney B., Lönnerdal B. (2011). Proteomic characterization of human milk whey proteins during a twelve-month lactation period. J. Proteome Res..

[38] Lönnerdal B., Erdmann P., Thakkar S.K., Sauser J., Destaillats F. (2017). Longitudinal evolution of true protein, amino acids and bioactive proteins in breast milk: A developmental perspective. J. Nutr. Biochem..

[39] Czosnykowska-Łukacka M., Orczyk-Pawiłowicz M., Broers B., Królak-Olejnik B. (2019). Lactoferrin in human milk of prolonged lactation. Nutrients.

[40] Czosnykowska-Łukacka M., Lis-Kuberka J., Królak-Olejnik B., Orczyk-Pawiłowicz M. (2020). Changes in human milk immunoglobulin profile during prolonged lactation. Front. Pediatr..

[41] Araújo E.D., Gonçalves A.K., Cornetta Mda C., Cunha H., Cardoso M.L., Morais S.S., Giraldo P.C. (2005). Evaluation of the secretory immunoglobulin A levels in the colostrum and milk of mothers of term and pre-term newborns. Braz. J. Infect. Dis..

[42] Chirico G., Marzollo R., Cortinovis S., Fonte C., Gasparoni A. (2008). Antiinfective properties of human milk. J. Nutr..

[43] Manzoni P. (2016). Clinical Benefits of Lactoferrin for Infants and Children. J. Pediatr..

[44] Donovan S.M. (2016). The role of lactoferrin in gastrointestinal and immune development and function: A preclinical perspective. J. Pediatr..

[45] Pammi M., Suresh G. (2017). Enteral lactoferrin supplementation for prevention of sepsis and necrotizing enterocolitis in preterm infants. Cochrane Database Syst. Rev..

[46] Gopalakrishna K.P., Hand T.W. (2020). Influence of maternal milk on the neonatal intestinal microbiome. Nutrients.

[47] Demers-Mathieu V., Mathijssen G., Dapra C., Do D.M., Medo E. (2020). Active free secretory component and secretory IgA in human milk: Do maternal vaccination, allergy, infection, mode of delivery, nutrition and active lifestyle change their concentrations?. Pediatr. Res..

[48] Ward P., Paz E., Conneely O. (2005). Lactoferrin. Cell. Mol. Life Sci..

[49] Lönnerdal B. (2016). Bioactive proteins in human milk: Health, nutrition, and implications for infant formulas. J. Pediatr..

[50] Drago-Serrano M.E., Campos-Rodríguez R., Carrero J.C., de la Garza M. (2017). Lactoferrin: Balancing ups and downs of inflammation due to microbial infections. Int. J. Mol. Sci..

[51] Redwan E.M., Uversky V.N., El-Fakharany E.M., Al-Mehdar H. (2014). Potential lactoferrin activity against pathogenic viruses. C. R. Biol..

[52] Lönnerdal B. (2009). Nutritional roles of lactoferrin. Curr. Opin. Clin. Nutr. Metab. Care.

[53] Vogel H.J. (2012). Lactoferrin, a bird’s eye view. Biochem. Cell Biol..

[54] Suzuki Y.A., Shin K., Lonnerdal B. (2001). Molecular cloning and functional expression of a human intestinal lactoferrin receptor. Biochemistry.

[55] Liao Y., Jiang R., Lonnerdal B. (2012). Biochemical and molecular impacts of lactoferrin on small intestinal growth and development during early life. Biochem Cell Biol..

[56] Butler J.E., Rainard P., Lippolis J., Salmon H., Kacskovics I. (2015). The mammary gland in mucosal and regional immunity. Mucosal Immunol..

[57] Goldman A.S., Chheda S., Garofalo R., Schmalstieg F.C. (1996). Cytokines in human milk: Properties and potential effects upon the mammary gland and the neonate. J. Mammary Gland Biol. Neoplasia.

[58] McManaman J.L., Neville M.C. (2003). Mammary physiology and milk secretion. Adv. Drug Deliv. Rev..

[59] Ballard O., Morrow A.L. (2013). Human milk composition: Nutrients and bioactive factors. Pediatr. Clin. N. Am..

[60] Liu B., Newburg D.S. (2013). Human milk glycoproteins protect infants against human pathogens. Breastfeed Med..

[61] Peterson R., Cheah W.Y., Grinyer J., Packer N. (2013). Glycoconjugates in human milk: Protecting infants from disease. Glycobiology.

[62] Lis-Kuberka J., Orczyk-Pawiłowicz M. (2015). The significance of fucosylated glycoconjugates of human milk in nutrition of newborns and infants. Pos. Hig. Med. Dosw. (Online).

[63] Bollinger R.R., Everett M.L., Palestrant D. (2003). Human secretory immunoglobulin A may contribute to biofilm formation in the gut. Immunology.

[64] Lönnerdal B. (2013). Bioactive proteins in breast milk. J. Paediatr. Child Health..

[65] Rogier E., Frantz A., Bruno M., Wedlund L., Cohen D.A., Stromberg A.J., Kaetzel C.S. (2014). Lessons from mother: Long-term impact of antibodies in breast milk on the gut microbiota and intestinal immune system of breastfed offspring. Gut Microbes..

[66] Hurley W.L., Theil P.K. (2011). Perspectives on immunoglobulins in colostrum and milk. Nutrients.

[67] Broadhurst M., Beddis K., Black J., Henderson H., Nair A., Wheeler T. (2015). Effect of gestation length on the levels of five innate defence proteins in human milk. Early Hum. Dev..

[68] Shashiraj Faridi M.M.A., Singh O., Rusia U. (2006). Mother’s iron status, breastmilk iron and lactoferrin—Are they related?. Eur. J. Clin. Nutr..

[69] Mastromarino P., Capobianco D., Campagna G., Laforgia N., Drimaco P., Dileone A., Baldassarre M.E. (2014). Correlation between lactoferrin and beneficial microbiota in breast milk and infant’s feces. Biometals.

[70] Trend S., Strunk T., Lloyd M.L., Kok C.H., Metcalfe J., Geddes D.T., Lai C.T., Richmond P., Doherty D.A., Simmer K. (2016). Levels of innate immune factors in preterm and term mothers’ breast milk during the 1st month postpartum. Br. J. Nutr..

[71] Yang Z., Jiang R., Chen Q., Wang J., Duan Y., Pang X., Jiang S., Bi Y., Zhang H., Lönnerdal B. (2018). Concentration of lactoferrin in human milk and its variation during lactation in different Chinese populations. Nutrients.

[72] Goonatilleke E., Huang J., Xu G., Wu L., Smilowitz J.T., German J.B., Lebrilla C.B. (2019). Human milk proteins and their glycosylation exhibit quantitative dynamic variations during lactation. J. Nutr..

[73] Marquis G.S., Penny M.E., Zimmer J.P., Díaz J.M., Marín R.M. (2003). An overlap of breastfeeding during late pregnancy is associated with subsequent changes in colostrum composition and morbidity rates among Peruvian Infants and their mothers. J. Nutr..

[74] Houghton M.R., Gracey M., Burke V., Bottrell C., Spargo R.M. (1985). Breast milk lactoferrin levels in relation to maternal nutritional status. J. Pediatr. Gastroenterol. Nutr..

[75] Leelahakul V., Tanaka F., Sinsuksai N., Vichitsukon K., Pinyopasakul W., Kido N., Inukai S. (2009). Comparison of the protein composition of breast milk and the nutrient intake between Thai and Japanese mothers. Nurs. Health Sci..

[76] Lis-Kuberka J., Orczyk-Pawiłowicz M., Królak-Olejnik B., Berghausen-Mazur M., Barańska K., Kątnik-Prastowska I. (2018). Lectin-based analysis of human milk immunoglobulin G fucosylated variants in relation to milk maturation and perinatal risk factors. J. Appl. Biomed..

[77] Koenig A., de Albuquerque Diniz E.M., Barbosa S.F.C., Vaz F.A.C. (2005). Immunologic factors in human milk: The effects of gestational age and pasteurization. J. Hum. Lact..

[78] Abuidhail J., Al-Shudiefat A.A., Darwish M. (2019). Alterations of immunoglobulin G and immunoglobulin M levels in the breast milk of mothers with exclusive breastfeeding compared to mothers with non-exclusive breastfeeding during months postpartum: The Jordanian cohort study. Am. J. Hum. Biol..

[79] Berdi M., de Lauzon-Guillain B., Forhan A., Castelli F.A., Fenaille F., Charles M.A., Heude B., Junot C., Adel-Patient K., EDEN Mother-Child Cohort Study Group (2019). Immune components of early breastmilk: Association with maternal factors and with reported food allergy in childhood. Pediatr. Allergy Immunol..

[80] Peila C., Gazzolo D., Bertino E., Cresi F., Coscia A. (2020). Influence of diabetes during pregnancy on human milk composition. Nutrients.

[81] Expert Committee on the Diagnosis and Classification of Diabetes Mellitus (2003). Report of the expert committee on the diagnosis and classification of diabetes mellitus. Diabetes Care.

[82] Wender-Ożegowska E., Bomba-Opońl D., Brązert J., Celewicz Z., Czajkowski K., Gutaj P., Malinowska-Polubiec A., Zawiejska A., Wielgoś M. (2018). Standards of Polish Society of Gynecologists and Obstetricians in management of women with diabetes. Ginekol. Pol..

[83] Araszkiewicz A., Bandurska-Stankiewicz E., Budzyński A., Cypryk K., Czech A., Czupryniak L. (2020). Guidelines on the management of diabetic patients. A position of Diabetes Poland. Clin. Diabetol..

[84] Wesolowska A., Sinkiewicz-Darol E., Barbarska O., Strom K., Rutkowska M., Karzel K., Rosiak E., Oledzka G., Orczyk-Pawiłowicz M., Rzoska S. (2018). New achievements in high-pressure processing to preserve human milk bioactivity. Front. Pediatr..

[85] Fuentes L., Roszer T., Ricote M. (2010). Inflammatory mediators and insulin resistance in obesity: Role of nuclear receptor signaling in macrophages. Mediators Inflamm..

[86] Sakowicz-Burkiewicz M., Pawelczyk T. (2011). Recent advances in understanding the relationship between adenosine metabolism and the function of T and B lymphocytes in diabetes. J. Physiol. Pharmacol..

[87] Badillo-Suárez P.A., Rodríguez-Cruz M., Nieves-Morales X. (2017). Impact of metabolic hormones secreted in human breast milk on nutritional programming in childhood obesity. J. Mammary Gland Biol. Neoplasia.

[88] Gallacher S.J., Thomson G., Fraser W.D., Fisher B.M., Gemmell C.G., MacCuish A.C. (1995). Neutrophil bactericidal function in diabetes mellitus: Evidence for association with blood glucose control. Diabet. Med..

[89] Rubinstein R., Genaro A., Motta A., Cremaschi G., Wald M. (2008). Impaired immune responses in streptozotocin-induced type I diabetes in mice. Involvement of high glucose. Clin. Exp. Immunol..

[90] Jafar N., Edriss H., Nugent K. (2016). The effect of short-term hyperglycemia on the innate immune system. Am. J. Med. Sci..

[91] Hu Y., Huang K., Sun Y., Wang J., Xu Y., Yan S., Zhu P., Tao F. (2017). Placenta response of inflammation and oxidative stress in low-risk term childbirth: The implication of delivery mode. B.M.C. Pregnancy Childbirth.

[92] Gardner H., Kent J.C., Lai C.T., Geddes D.T. (2019). Comparison of maternal milk ejection characteristics during pumping using infant-derived and 2-phase vacuum patterns. Int. Breastfeed. J..

[93] Kaushik S., Trivedi S.S., Jain A., Bhattacharjee J. (2002). Unusual changes in colostrum composition in lactating indian women having medical complications during pregnancy—A pilot study. Indian J. Clin. Biochem..

[94] Morceli G., Franca E., Magalha˜es V., Damasceno D., Calderon I., Honorio-Franca A. (2011). Diabetes induced immunological and biochemical changes in human colostrum. Acta Paediatr..

[95] Franca E.L., Calderon I.M.P., Vieira E.L., Morceli G., Honorio-Franca A.C. (2012). Transfer of maternal immunity to newborns of diabetic mothers. Clin. Dev. Immunol..

[96] Smilowitz J.T., Totten S.M., Huang J., Grapov D., Durham H.A., Lammi-Keefe C.J., Lebrilla C., German J.B. (2013). human milk secretory immunoglobulin A and lactoferrin N-glycans are altered in women with gestational diabetes mellitus. J. Nutr..

[97] Ali A.M., Kunugi H. (2020). Intermittent fasting, dietary modifications, and exercise for the control of gestational diabetes and maternal mood dysregulation: A review and a case report. Int. J. Environ. Res. Public Health.

[98] Demers-Mathieu V., Huston R.K., Markell A.M., McCulley E.A., Martin R.L., Spooner M., Dallas D.C. (2019). Differences in maternal immunoglobulins within mother’s own breast milk and donor breast milk and across digestion in preterm infants. Nutrients.

[99] Woof J.M., Russell M.W. (2011). Structure and function relationships in IgA. Mucosal Immunol..

[100] Koch M.A., Reiner G.L., Lugo K.A., Kreuk L.S., Stanbery A.G., Ansaldo E., Seher T.D., Ludington W.B., Barton G.M. (2016). Maternal IgG and IgA antibodies dampen mucosal T helper cell responses in early life. Cell.

[101] Gomez de Aguero M., Ganal-Vonarburg S.C., Fuhrer T., Rupp S., Uchimura Y., Li H., Steinert A., Heikenwalder M., Hapfelmeier S., Sauer U. (2016). The maternal microbiota drives early postnatal innate immune development. Science.

[102] Lawrence R.M., Pane C.A. (2007). Human breast milk: Current concepts of immunology and infectious diseases. Curr. Probl. Pediatr. Adolesc. Health Care.

[103] Brandtzaeg P. (2010). The mucosal immune system and its integration with the mammary glands. J. Pediatr..

[104] Zeng M.Y., Cisalpino D., Varadarajan S., Hellman J., Warren H.S., Cascalho M., Inohara N., Núñez G. (2016). Gut microbiota-induced immunoglobulin G controls systemic infection by symbiotic bacteria and pathogens. Immunity.

[105] Caballero-Flores G., Sakamoto K., Zeng M.Y., Wang Y., Hakim J., Matus-Acuña V., Inohara N., Núñez G. (2019). Maternal immunization confers protection to the offspring against an attaching and effacing pathogen through delivery of IgG in breast milk. Cell Host Microbe.

[106] Zheng W., Zhao W., Wu M., Song X., Caro F., Sun X., Gazzaniga F., Stefanetti G., Oh S., Mekalanos J.J. (2020). Microbiota-targeted maternal antibodies protect neonates from enteric infection. Nature.

[107] Royle L., Roos A., Harvey D.J., Wormald M.R., van Gijlswijk-Janssen D., Redwan E.-R.M., Wilson I.A., Daha M.R., Dwek R.A., Rudd P.M. (2003). Secretory IgA N- and O-glycans provide a link between the innate and adaptive immune systems. J. Biol. Chem..

[108] Lis-Kuberka J., Królak-Olejnik B., Berghausen-Mazur M., Orczyk-Pawiłowicz M. (2019). Lectin-based method for deciphering human milk IgG sialylation. Molecules.

[109] Pace R.M., Williams J.E., Järvinen K.M., Belfort M.B., Pace C.D., Lackey K.A., Gogel A.C., Nguyen-Contant P., Kanagaiah P., Fitzgerald T. (2020). COVID-and human milk: SARS-CoV-2, antibodies, and neutralizing capacity. medRxiv.

[110] Ruiz L., Espinosa-Martos I., García-Carral C., Manzano S., McGuire M.K., Meehan C.L., McGuire M.A., Williams J.E., Foster J., Sellen D.W. (2017). What’s normal? Immune profiling of human milk from healthy women living in different geographical and socioeconomic settings. Front Immunol..

[111] Moirasgenti M., Doulougeri K., Panagopoulou E., Theodoridis T. (2019). Psychological stress reduces the immunological benefits of breast milk. Stress Health..

[112] Walters D.D., Phan L.T.H., Mathisen R. (2019). The cost of not breastfeeding: Global results from a new tool. Health Policy Plan..

